# Long-Term Follow-Up of a Severe Eosinophilic Asthmatic Patient With Comorbid Nasal Polyposis Hospitalized for SARS-CoV-2 Infection While Receiving Benralizumab: A Case Report

**DOI:** 10.7759/cureus.20364

**Published:** 2021-12-12

**Authors:** Annamaria Ambrosino

**Affiliations:** 1 Pneumology, S. Maria Delle Grazie Hospital, Pozzuoli, ITA

**Keywords:** long term follow up, "anti il5-r", "emergency visit" "spirometry" "biologic" "long term follow up", emergency medical service, "covid-19" "severe eosinophilic asthma" "nasal polyps" "benralizumab" "interstitial pneumonia"

## Abstract

We report a case of a patient affected by severe eosinophilic asthma with nasal polyps (SEA+NP) who developed coronavirus disease 2019 (COVID-19) six months after starting benralizumab as add-on therapy. Both SEA and NP were under control with no exacerbations at the time of severe acute respiratory syndrome coronavirus 2 (SARS-CoV-2) infection. The patient was hospitalized for four months, during which the treatment with benralizumab was interrupted. Despite the onset of bilateral interstitial pneumonia, developed as a consequence of the SARS-CoV-2 infection, the patient was discharged without complications, with a significant improvement in the chest CT scan following the administration of systemic corticosteroids (SCS) and low-flow oxygen therapy. The treatment with benralizumab was reintroduced at the regular dosing regimen immediately after his discharge. Lung function was assessed three months after the discharge and showed normal levels as before the development of COVID-19 symptoms. A long-term follow-up after 26 months from the introduction of benralizumab showed a normal lung function and well-controlled asthma, without exacerbations or the need for corticosteroid bursts.

## Introduction

Severe acute respiratory syndrome coronavirus 2 (SARS-CoV-2), which causes coronavirus disease 2019 (COVID-19), has been recognized as a highly pathogenic virus that infects the human respiratory tract and leads to high morbidity and mortality [1-3]. Underlying conditions such as chronic respiratory diseases may affect the progression, treatment, and prognosis of COVID-19 [4-6]. Patients affected by severe asthma are at greater risk of serious illness if infected [7]. Hence, the management of patients with severe asthma during the coronavirus pandemic has come under intense scrutiny and debate. Current evidence-based guidelines highlight the importance of maintaining asthma control and avoiding exacerbation risks [8]. However, indications for the management of severe asthma in patients who contracted COVID-19 and are being treated with monoclonal antibodies prior to and after infection with COVID-19 are poor. The current practice is to continue with the administration of biological therapies during the COVID-19 pandemic in patients with asthma for whom such therapies are indicated and have been effective [8]. For patients with severe asthma infected by SARS-CoV-2, the decision to maintain or postpone the biological therapy, until they recover, should be made on a case-by-case basis with the aid of a multidisciplinary team [9].

## Case presentation

**Figure 1:**
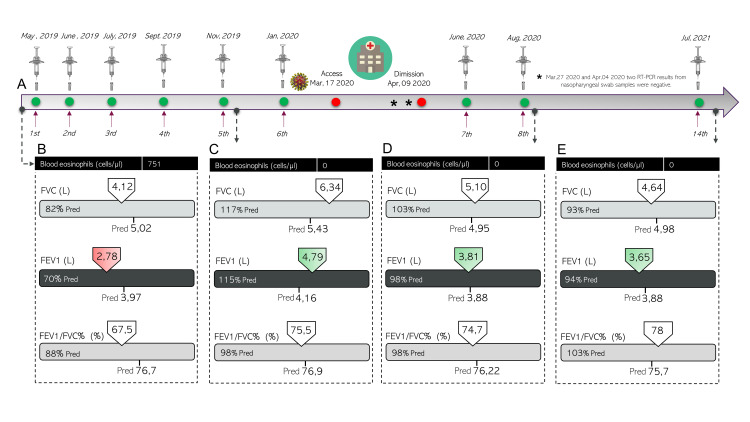
Case summary A) Schedule of benralizumab administration and suspension due to SARS-CoV-2-related hospitalization. B) Lung function parameters at baseline, before benralizumab treatment. C) Lung function parameters after six months of therapy with benralizumab and before SARS-CoV-2 infection. D) Lung function parameters after SARS-COV-2 infection and hospitalization. E) Lung function parameters at the two-year follow-up SARS-CoV-2: severe acute respiratory syndrome coronavirus 2; FVC: forced vital capacity; FEV1: forced expiratory volume after the first second; pred: predicted; RT-PCR: reverse transcription-polymerase chain reaction

In this report, we present the case of a 59-year-old man diagnosed with severe eosinophilic asthma with nasal polyps (SEA+NP), with no other comorbidities. Treatment with benralizumab, an anti-interleukin-5 receptor alpha monoclonal antibody [10], started in May 2019 (Figure 1a) due to poor asthma control and frequent severe exacerbations requiring systemic corticosteroids (SCS) despite the use of controller therapies consisting of high-dose inhaled corticosteroids (ICS), inhaled long-acting beta-agonists (LABA), and once-daily montelukast. In particular, in April 2019, the patient's blood eosinophil count (BEC) had been 751 cells/mm^3^ and spirometric evaluation had revealed a pre-bronchodilator forced expiratory volume after one second (FEV1) of 2.78 L (70% of predicted) (Figure 1b). After six months of benralizumab treatment (administered for a total of five doses, the first three doses given every four weeks, and then every eight weeks), the patient showed a rapid improvement in asthma control and NP symptoms and did not experience any exacerbations requiring SCS use. A significant improvement in FEV1 was also registered (4.16 L, 115% of predicted) (Figure 1c).

On March 7, 2020, the patient experienced flu-like symptoms, characterized by persistent moderate fever, dry cough, and respiratory distress. After 10 days, a nasopharyngeal swab for SARS-CoV-2 was performed and returned positive. Home-based COVID-19 treatment with azithromycin 300 mg was started and the patient was thereafter admitted to ER on March 17 due to the worsening of his symptoms.

**Figure 2:**
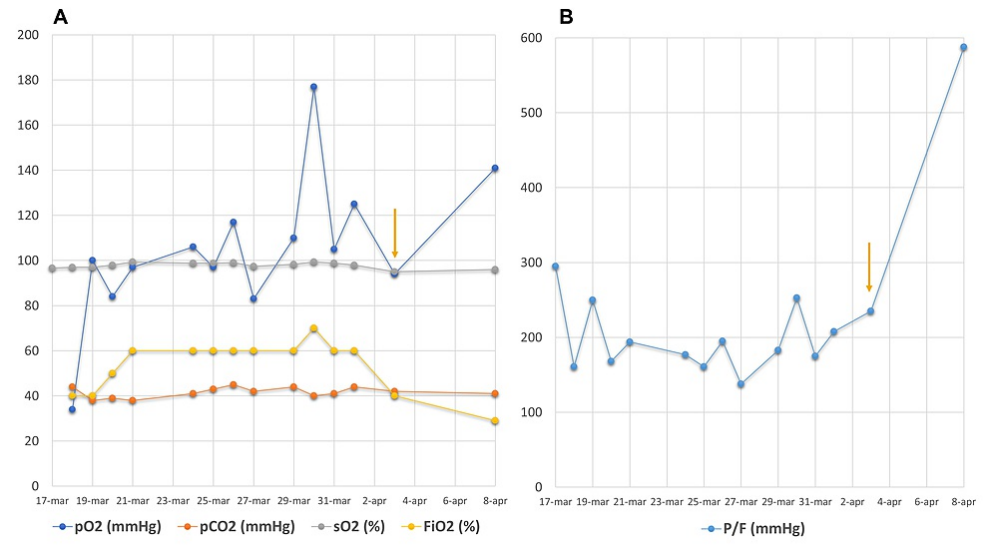
Blood gas analysis during hospitalization A) Variation of pO_2_, pCO_2_, and sO_2_ during hospitalization. B) Variation of P/F ratio during hospitalization Golden arrows indicate the time of SCS introduction pO_2_: partial pressure of oxygen; pCO_2_: partial pressure of carbon dioxide; sO_2_: oxygen saturation; P/F: Horowitz index for lung function, calculated as the partial pressure of oxygen divided by the fraction of inspired oxygen; SCS: systemic corticosteroids

At the time of ER admission, borderline hypercapnia with respiratory failure was found (Figure 2a).

**Figure 3:**
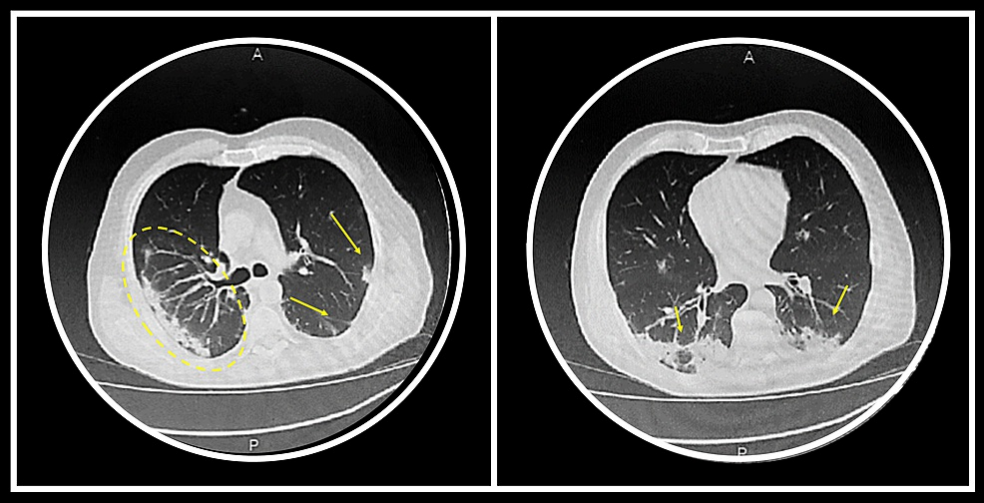
Chest CT during hospital admission Consolidations and ground-glass opacities with prevalent sub-pleural distribution are shown (yellow circle). Multiple small ground-glass areas are present in both lungs, with the same distribution (arrows). CT scans are consistent with COVID-19 interstitial pneumonia CT: computed tomography; COVID-19: coronavirus disease 2019

CT scan of the lungs revealed bilateral consolidation and ground-glass opacities, consistent with COVID-19 pneumonia (Figure 3).

Laboratory tests (Table 1) showed elevated markers of inflammation, with C-reactive protein (CRP), erythrocyte sedimentation rate (ESR), and ferritin above the normal limits. The slight neutrophilia was not related to bacterial infection, as procalcitonin returned negative. BEC was 0 cells/mm^3^, in line with benralizumab treatment. A slight increase in liver enzymes and both direct and total bilirubin levels were found. No clear signs of macrophage activation syndrome were present (no increase in triglyceride levels, normal fibrinogen levels).

**Table 1:** Main laboratory tests BUN: blood urea nitrogen; AST: aspartate aminotransferase; ALT: alanine transaminase; GGT: gamma-glutamyl transferase; LDH: lactic acid dehydrogenase; HDL: high-density lipoprotein; LDL: low-density lipoprotein; CK: creatine kinase; ESR: erythrocyte sedimentation rate; CRP: C-reactive protein; BNP: brain natriuretic peptide

Parameters	Normal range	March 17	April 1	April 8
Lymphocytes (%)	(20-40)	20.6	25.4	12.8
Monocytes (%)	(2-6)	7.2	13.9	6.6
Neutrophils (%)	(50-70)	71.9	60.3	80.5
Eosinophils (%)	(0-4)	0	0	0
Basophils (%)	(0-1)	0.3	0.4	0.1
Creatinine (mg/dL)	(0.6-0.9)	0.9	0.8	0.6
BUN (mg/dL)	(10-50)	22	25	34
Uric acid (mg/dL)	(3.7-9.2)	4	3	2.3
Sodium (mEq/L)	(135-145)	140	140	141
Potassium (mEq/L)	(3.5-5.1)	3.9	4.5	4.5
Glucose (mg/dL)	(74-106)	127	85	114
AST (UI/L)	(0-34)	54	26	31
ALT (UI/L)	(10-49)	46	54	85
GGT (UI/L)	(7-73)	145	98	105
Cholinesterase (UI/L)	(7,000-19,000)	9,817	8,124	9,476
Fractionated total bilirubin (mg/dL)	(0.2-1)	2.2	0.6	0.5
Direct bilirubin (mg/dL)	(0.00-0.2)	1.3	0.3	0.2
Total protein (g/dL)	(5.7-8.2)	6.5	5.2	5.8
Albumin (g/dL)	(3.2-4.8)	4.2	3.3	3.8
LDH (UI/L)	(100-246)	248	178	180
Amylase (UI/L)	(25-115)	40	91	70
Lipase (UI/L)	(12-53)	44	61	47
Triglycerides (mg/dL)	(50-150)	140	223	83
Cholesterol (mg/dL)	(<200)	105	100	164
HDL cholesterol (mg/dL)	(40-60)	26	25	45
LDL cholesterol (mg/dL)	(50-129)	53	47	115
CK (UI/L)	(46-171)	45		18
Myoglobin (ng/dL)	(14-106)	80	38	25
ESR (mm, 1 hour)	(2-14)	65	38	15
CRP (mg/dL)	(<1.0)	7.6	<0.4	<0.4
Ferritin (ng/ml)	(10-450)	730	540.8	530.3
Iron (µg/dL)	(65-175)		101	92
Fibrinogen (mg/dL)	(175-417)		363	247
Interleukin-6 (pg/mL)	(0-5)		4.7	
BNP (pg/ml)	(<100)		2	16
High sensitivity troponin I (pg/mL)	(40.8-115.1)		<2.5	<2.5
D-dimer (ng/mL)	(<250)		400	214
Antithrombin III (%)	(75-125)			106
Procalcitonin (ng/mL)	(<0.5)	0.05		<0.02

**Figure 4:**
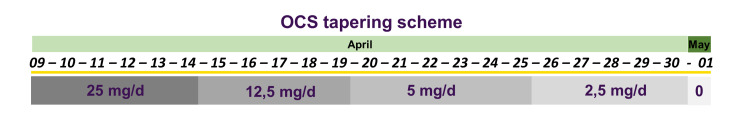
Prednisone tapering scheme after hospital discharge OCS: oral corticosteroids

Oxygen support (10 L/minute) was provided, and treatment with ritonavir+lopinavir 200/50 mg, hydroxychloroquine 200 mg, amoxicillin/clavulanate 850/150 mg twice daily, azithromycin 300 mg twice daily, omeprazole 20 mg, and paracetamol 1000 mg was started. Benralizumab administration was interrupted since there were no clear indications to continue with the therapy at that time. Due to substantial stability of the clinical and laboratory parameters, anti-viral and antibiotic treatments were suspended after 17 days, and therapy with enoxaparin 4000 UI/day and prednisone 25 mg daily was started, with concomitant amelioration of the P/F ratio (Figure 2b). The patient was discharged on April 9 with the recommendation to continue the treatment with prednisone at 25 mg/day for one week, to be tapered off according to the scheme reported in Figure 4.

Benralizumab administration was resumed in July 2020 with the standard eight-week scheme (Figure 1a). On September 7, after two additional doses of benralizumab, lung function parameters returned to normal levels (Figure 1d). In July 2021, one year and three months after discharge and recovery from COVID-19, and 26 months after benralizumab introduction, the patient had a completely controlled asthma, with no exacerbation or SCS use recorded, and lung function parameters were still found to be within normal levels (Figure 1e).

## Discussion

We presented the case of a male patient contracting COVID-19 while on benralizumab for the treatment of severe asthma. To our knowledge, this is the first report about the long-term follow-up (26 months) of a SEA+NP patient infected with SARS-CoV-2 while on treatment with benralizumab with lung function parameters measured before and after the development of COVID-19 symptoms.

The course of the disease was mildly severe, requiring the administration of SCS. The control of asthma and the stability of the conditions achieved before the SARS-CoV-2 infection might have contributed to a favorable COVID-19 prognosis in this patient. The same observation has also been made in other studies, where the severity of COVID-19 was found to be positively associated with the number of asthma exacerbations in patients treated with monoclonal antibodies [11]. Even though eosinopenia has been considered a marker of SARS-CoV-2 infection and is believed to aggravate the severity of the disease [12], more recent reports have failed to confirm these data [13]. In our case report, eosinopenia caused by benralizumab did not worsen the overall condition. On the contrary, our patient experienced an improvement in lung function parameters, which reached the same levels as before contracting COVID-19. The improvement showed by our patient appears to be in contrast with previous literature, which indicates that patients recovering from COVID-19 do not completely recover their lung function [14]. The most probable reason for this discrepancy is that our patient was treated with benralizumab, which had been started to be administered before the patient contracted COVID-19; it was suspended during hospitalization and mechanical ventilation and was resumed immediately after his discharge. Another point of interest is that contrary to the case report by Kroes et al. [15], benralizumab administration was suspended during hospitalization and mechanical ventilation. Overall, in our patient, the suspension of benralizumab for five months and its reintroduction with the classic eight-week scheme did not cause any loss of efficacy of the biologic treatment: no exacerbation or SCS use was recorded and lung function parameters were re-established to normal levels, indicating a good control of asthma.

## Conclusions

This case study describes for the first time the favorable outcome in a SEA+NP patient treated with benralizumab after contracting SARS-CoV-2 infection, with a follow-up of two years. Before the initiation of the treatment with benralizumab, the patient had poor asthma control, impaired lung function, and frequent severe exacerbations requiring SCS. The improvement in lung function achieved with benralizumab was maintained after admission and remained stable over a year after hospital admission, supporting the efficacy, safety, and durability of treatment with benralizumab in patients affected by COVID-19.
